# The diagnostic value of transcranial sonography in Swedish parkinsonism patients: A retrospective cohort study with long-term follow-up

**DOI:** 10.1016/j.prdoa.2025.100411

**Published:** 2025-12-06

**Authors:** M. Stiehm, C. Nilsson, Ö. Skogar, U. Walter

**Affiliations:** a Dept. of Neurology, Skåne University Hospital, Malmö/Lund, Sweden; b Cognitive Disorder Research Unit, Dept. of Clinical Sciences, Lund University, Lund, Sweden; c Dept. of Clinical Sciences, Lund University, Lund, Sweden; d Dept. of Neurology, Rostock University Medical Center, Rostock, Germany; e German Center for Neurodegenerative Diseases (DZNE) Rostock/Greifswald, Rostock, Germany

**Keywords:** Transcranial sonography, Parkinsonism, Ultrasound, Parkinsońs disease, Substantia nigra, Hyperechogenicity, Movement disorders, Essential tremor

## Abstract

**Introduction:**

Although transcranial sonography (TCS) assessing hyperechogenic substantia nigra (SN+) as biomarker for Parkinsońs disease (PD) has been introduced elsewhere, the clinical relevance and accuracy in a Swedish population is still unknown.

**Methods:**

This retrospective single-center study included 74 patients with predominantly early-stage parkinsonism at first visit who had been examined by TCS from 2013 to 2017 to determine the SN+ biomarker status in relation to PD, atypical parkinsonian disorders (APS), essential tremor (ET) and vascular/secondary/ unspecified parkinsonism, with the aim of long-term follow-up to confirm the clinical diagnosis. The cut-off value for SN+ was regarded as the 90 % percentile of SN echogenicity in a local healthy cohort (here, 0.23 cm^2^).

**Results:**

In 2024, the mean follow-up time was 95 months. Three patients (4 %) without transcranial bone were excluded. SN+ was found in 38/51 patients with finally diagnosed PD and 4/20 patients with other final clinical diagnoses (p < 0.001). Sensitivity was moderate (75 %) whereas specificity and the positive predictive value were higher (80 % and 90 %, respectively). SN area measurements (most abnormal side) were significantly different in PD-patients compared to non-PD patients (n = 63; 0.28 ± 0.09 [95 % CI: 0.25–0.30] cm^2^ vs. 0.23 ± 0.10 [0.18–0.29] cm^2^, p 0.035).

**Conclusion:**

After a follow-up of up to 8 years, to maximize diagnostic certainty, our findings support the use of TCS as a valuable add-on tool in PD diagnostics in a Swedish patient population, already in the early stage of disease but not for screening.

## 1 Introduction

Basal ganglia disorders such as Parkinson's disease (PD), essential tremor (ET) and atypical parkinsonian syndromes (APS) are of increasing relevance to the society due to an ageing population [1,2]. PD is the second-most common neurodegenerative disease with a prevalence of over 22,000 patients in Sweden and entails significant societal costs [3]. Symptom onset in PD seldom occurs before the age of 50 and the incidence increases with age [1]. The diagnosis is made clinically by patient interview and physical examination, no single technical examination can currently replace the doctor's assessment. APS include multisystem atrophy (MSA), progressive supranuclear palsy (PSP) and corticobasal degeneration (CBD). Vascular parkinsonism (VP) is regarded as a secondary parkinsonian syndrome. In recent years, nuclear medicine methods using SPECT or PET have been applied to supplement clinical diagnostics, but they are limited by cost and availability and cannot always reliably differentiate between different parkinsonian syndromes.

Transcranial sonography (TCS) is a non-invasive, cheap, fast and easily applicable but partially operator-dependent imaging technique that could be used to support a PD diagnosis and to distinguish PD from APS [4,5] and ET. There are no known side effects of TCS. Around 80 % of patients with idiopathic PD but only about 10 % of a healthy population show a markedly increased echogenicity (hyperechogenicity) of substantia nigra (SN) when examined by TCS [6], probably due to pathological iron accumulation [7]. Several previous studies in a Central European population indicate that SN hyperechogenicity (SN+) itself is a rather stable biomarker regardless of how long the patient has suffered from PD [8]. Some studies show that SN+ status could have added value in the diagnosis of PD, especially in younger patients, even before the first motor symptoms appear [9]. SN is measured by semi-quantitative visual assessment and planimetric area measurement ipsilaterally by transtemporal B-mode ultrasound if there is a good or satisfactory bone window [10]. Moderate SN+ refers to planimetric areas larger than 75 % percentile in adult population without neurological disorders, whereas marked SN+ refers to areas larger than 90 % percentile [4]. The risk of clinically asymptomatic individuals over the age of 50 developing PD is up to 20 times higher in the presence of SN+ [11,12]. Previously, a significant correlation between FDG-PET and TCS findings was demonstrated in the differentiation of Parkinson's disease [13]. Studies with a follow-up period of up to 5 years showed that TCS is particularly beneficial in the early stages of PD, when patients only have mild extrapyramidal symptoms. In cases where APS was suspected, the studies were smaller and the follow-up period was significantly shorter, at 12 to 17 months [12,[14], [15], [16]]. Since 2013, the European neurological societies have included TCS for the differential diagnosis of PD, and optionally for screening persons at risk of developing PD (level A recommendation) [17,18]. The available studies were performed in Central European, East Asian and South American populations with relatively short follow-up time (for PD: range 0–60 months; for APS: 0–17 months). So far, however, there are no trials on the use of TCS in a Scandinavian population.

The aim of this study was to evaluate the diagnostic value of the TCS finding SN+ in newly diagnosed patients with early-stage parkinsonism with regard to the final diagnosis (PD vs. non-PD, including APS, secondary/vascular parkinsonism, or ET) after long-term follow-up (FU) at our tertiary center in southern Sweden.

## 2 Material & methods

### 2.1 Patients

From 2013, we started offering all newly referred and selected chronic patients with parkinsonism a TCS examination, as part of our routine clinical diagnostic workup. From 2015, we achieved a sustained high degree of patient participation.

**Fig. 1:**
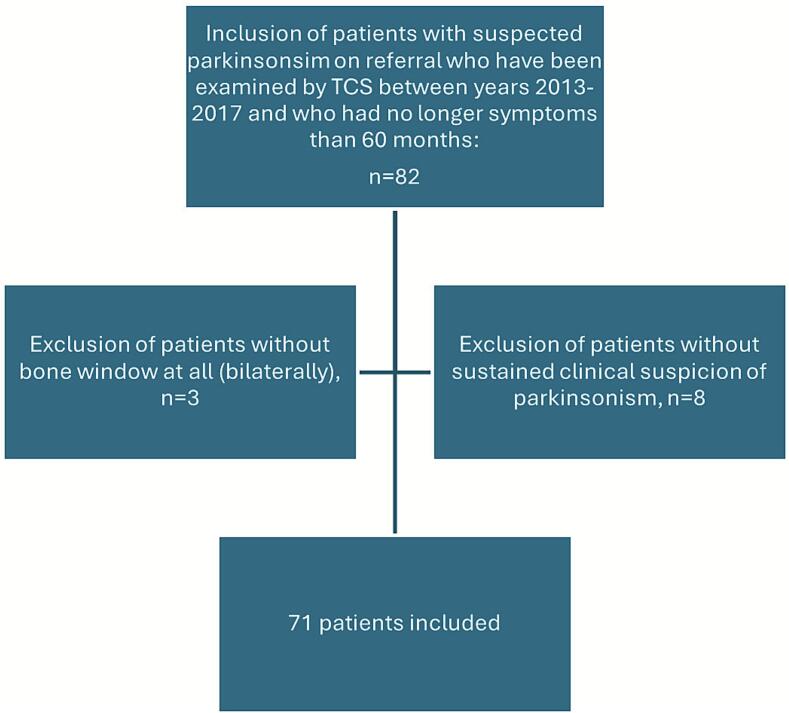
Flow chart regarding criteria for inclusion and exclusion of study participants.

The present study was conducted as a retrospective cohort study, see Fig. 1. New patients with parkinsonism who presented to our center before 2017 who have been examined by TCS during 2013 to 2017 (non-consecutive recruitment) could be included (n = 74). Patients with dementia that was already present at their first visit were not included. The duration of parkinsonian symptoms was equal with or less than five years at clinical examination in all study participants regardless of when they presented to our center. Among these, only three patients with unsatisfactory bone window were excluded. All included patients were assessed clinically by a specialist in neurology with extensive experience in movement disorders to establish clinical diagnosis.

### 2.2 Transcranial sonography (TCS)

TCS was performed transtemporally by an at that time commonly available high-resolution ultrasonic system (iU22; Philips, Eindhoven, The Netherlands). The following brain structures were depicted and assessed: substantia nigra and the ventricular system, especially the width of 3rd ventricle. For this, a 2- to 3-MHz ultrasound transducer was used for transtemporal axial examination of midbrain plane and thalamus plane in B-mode [4,19]. Dynamic range was set at 45–55 dB, depth at 14–16 cm so that the contralateral skull bone was visible. Other settings were adjusted as needed, with optional use of harmonic imaging as previously described [19]. After manually encircling the echogenic area of the SN with the cursor, the area was automatically calculated by the ultrasound device without the use of external software. Ultrasound findings were recorded digitally as images (see bonus figures A and B in the supplementary material) and short video loops on the machine, as summary report (text), and as measurement data collection in a regional clinical image database. All other study data and the measurements were saved in an external research database. For various ultrasound systems of different manufacturers, the cut-off values defining SN+ have been established by several research groups and clinical units [20]. In the present study, the first author is an experienced, board-certified neurosonologist. In an initial local validation study a group of voluntary healthy probands (n = 40; four without bone window; mean age: 44 ± 14 (mean ± SD, range, 25–71 years); sex: 27 women; mean SN echogenic area at left side: 0.133 ± 0.051 cm^2^, right side: 0.148 ± 0.073 cm^2^) were investigated on both sides by TCS planimetric measurements. In this reference population the measures corresponded well with previously reported data obtained with the same type of ultrasound system in a larger cohort of 105 healthy persons [21], and the 90 % percentile of SN echogenicity was determined at 0.23 cm^2^. Therefore, values > 0.23 cm^2^ were defined as marked SN+ in our population.

### 2.3 Clinical follow-up and final diagnoses of parkinsonian patients

The final clinical diagnosis was extracted from each patient’s medical records and confirmed by review of the medical records by a senior PD specialist with long-standing experience in movement disorders independently from the TCS investigator, complicated or unclear cases were discussed, and diagnoses were established by a three headed consensus group. The established MDS clinical diagnostic criteria for PD were applied [22]. The diagnosis could consider the results of dopaminergic nuclear medicine examinations if performed (SPECT or ^18^F-DOPA-PET). There was no evidence in the patient records of diagnostic bias or interference between the initial TCS findings and the final diagnosis, as the diagnosis was not changed by the treating neurologists directly or within one year of the TCS examination in any patient, even when the TCS results were documented and available in the medical records. Furthermore, TCS was not performed either before the initial diagnosis or during the first visit to our center.

### 2.4 Statistics and ethics

Statistical analyses were performed using IBM SPSS version 30.0. Numerical variables were tested by the non-parametric Mann-Whitney *U* test and categorical variables by the Chi^2^-test. The study was approved by the Swedish Ethical Review Authority (Dnr 2022–06697-01).

## 3 Results

Seventy-four patients were recruited after being examined by TCS at our center between years 2013–2017. The TCS data of 71 patients could be analyzed, as three patients had no sufficient bone window for TCS. Of these, 51 were finally diagnosed with PD and 20 with non-PD basal ganglia disorder (eight APS, two ET, seven with secondary/VP and three with unspecified parkinsonism). Thirty patients underwent additional dopamine transporter-imaging by SPECT and/or PET examination during FU (24 were abnormal). Duration of FU was in mean 95.3 ± 48.2 months (range 1–217). Patients were 66 ± 10.0 (range 43–88) years of age at the first visit and had a mean motor symptom duration of 18 months (range 4–60). Time from 1st visit to TCS examination for the whole group was in median 8.0 months (range 1–106), but for the 55 patients examined between 2015–2017 it was in median 6.0 months (range 1–87). 24 patients died and 17 patients developed dementia during FU. For summarized baseline characteristics, see Table 1.

**Table 1:** Baseline characteristics and diagnoses after follow-up. *Abbreviations: Follow-Up (FU); Transcranial Sonography (TCS); Parkinson s disease (PD); Atypical Parkinsonism (APS); Essential Tremor (ET)*.

Variable	All (n = 71)
*Demographics*	
Mean age (range)	66 (43–88)
Women/men	27/44
*Time variables*	
Mean FU duration in months (range)	95.3 (1–217)
Median symptom duration prior visit in months (range)	18 (4–60)
Median time from 1st visit to TCS examination in months (range)	8 (1–106)
*Medical*	
Change of diagnosis during FU, no. (%) incl. change after consensus group meeting	37 (52) 4 (6)
PET and SPECT, no. (%) of which - pathological, no. (%) - SWEDD*, no.	30 (41) 24 (34) 1
Died during FU, no. (%)	24 (34)
Dementia during FU, no. (%)	17 (24)
Final diagnosis after FU, no. (%)	
PD	51 (72)
APS	8 (11)
ET	2 (3)
Secondary incl. VP/unspecified parkinsonism	7/3 (14)

* : Scans without evidence of dopaminerg deficit (SWEDD).

There was a difference between the groups with regard to the SN+ biomarker, which occurred significantly more frequently in the PD group (p < 0.001), see Table 2. The descriptive statistics of the SN+ findings related to a PD diagnosis are presented in table 3 (supplementary material): SN+ was found in 38/51 finally diagnosed PD-patients (75 % sensitivity, 95 % CI 60–86 %) and 38/42 test-positive patients (SN+) had a final PD-diagnosis (90 % positive predictive value, 95 % CI: 80–96 %). The specificity was 80 % (56–94 %), the accuracy 76 % (64 %–85 %) and the negative predictive value 55 % (42 %–67 %).

**Table 2:** Hyperechogenicity of substantia nigra vs. final diagnosis. Abbreviations: hyperechogenicity of substantia nigra (SN+); Parkinson s disease (PD).

	PD (no.)	Non-PD (no.)	All (no.)	p
SN+	38	4	42	<0.001
Normal	13	16	29	
	51	20	71	

At FU, diagnosis had been changed in 52 % of cases, in either direction (any time-point after the first visit). For example, 5 (14 %) of 36 initially suspected PD cases had a final diagnosis of APS and eight of 16 patients with initial suspected secondary/unspecified basal ganglia disorder got a final diagnosis of PD. Two initially suspected PD cases were diagnosed with VP/secondary parkinsonism (out of in total seven with secondary/vascular parkinsonism) leaving only three patients with undefined parkinsonism after FU, see table 4 in the supplementary material for details. In the subgroup of revised patients, 17 of 22 true PD-patients demonstrated SN+ on TCS (vs. 3 of 15 non-PD-patients, p < 0.001) which corresponds to a 77 % sensitivity (95 % CI 55 %-92 %), 80 % specificity (95 % CI 52 %-96 %) and 85 % PPV (95 % CI 67 %-94 %). When excluding the patients with longer delay between symptom debut and TCS over 60 months (n = 13) the results did not change substantially, we still found a significant difference between groups (28 of 41 PD with SN+ vs. 4 of 17 non-PD with SN+; p 0.002) but lower values for sensitivity (68 %; 95 %CI 52 %-82 %), specificity (775 %; 95 % CI 50 %-93 %) and the positive predictive value (88 %; 95 % CI 74 %-94 %). Even when excluding all patients examined beyond 24 months from symptom onset, the smaller subgroup (n = 30) showed similar results: 13/21 PD patients were positive vs. 2/9 non-PD (p 0.046) which corresponds to 62 % sensitivity (95 % CI 38 %-82 %), 78 % specificity (95 % CI 40 %-97 %) and 87 % positive predictive value (95 % CI 65 %-96 %).

In addition, there was a clear difference in the subgroup of patients with dopamine scans: 10 of 15 PET/SPECT-positive PD patients showed the SN+ finding, while only 1 of 9 PET/SPECT-positive patients without clinical PD showed this finding on TCS (p = 0.008); this corresponds to a sensitivity of 67 % (95 % CI 38–88 %), a specificity of 89 % (95 % CI 52–100 %), and a PPV of 91 % (95 % CI 60–99 %) in this subgroup. Altogether, 30 scans were recorded in 26 patients, with 24 scans being pathological, one being inconclusive, and the rest being normal. SWEDD was detected in one patient.

**Fig. 2:**
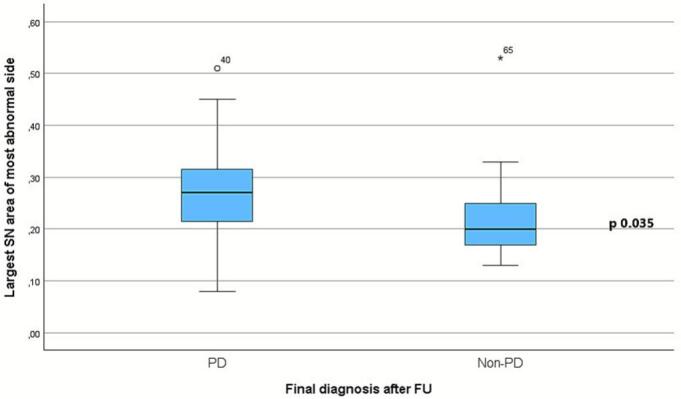
Boxplots of largest mean SN-size (cm^2^) of both sides (the most abnormal side) vs. final diagnosis PD or Non-PD (n = 63; 0.28 ± 0.09 [95 % CI: 0.25–0.30] cm^2^ vs. 0.23 ± 0.10 [0.18–0.29] cm^2^, p 0.035). *MWU-test.

Quantitative measures of SN echogenic area were available in 63 patients. The average size of SN echogenic area in PD-patients tended to be larger on each side as compared to the non-PD patients but that finding was non-significant (left side: 0.24 ± 0.11 [95 % CI: 0.20–0.27] cm^2^ vs. 0.20 ± 0.05 [0.17–0.23] cm^2^, p 0.156; right side: 0.26 ± 0.09 [0.23–0.29] cm^2^ vs. 0.21 ± 0.12 [0.13–0.29] cm^2^, p 0.123). The largest size of SN echogenic area of either left or right side was significantly different in PD-patients compared to non-PD patients (n = 63; 0.28 ± 0.09 [95 % CI: 0.25–0.30] cm^2^ vs. 0.23 ± 0.10 [0.18–0.29] cm^2^, p 0.035), see Fig. 2. No significant difference of the 3rd ventricle widths was found between 46 PD-patients (0.44 ± 0.19 [95 % CI: 0.38–0.49] cm) and 17 non-PD-patients (0.49 ± 0.27 [95 % CI: 0.35–0.64] cm; p 0.52) nor between PD and APS patients (0.47 ± 0.28 [95 % CI: 0.24–0.71] cm; n = 8; p 0.91).

## 4 Discussion

Although TCS of the SN echo signals has been studied since many years and has been recommended as a diagnostic tool in European und National clinical guidelines on PD [17,18], it is worth re-examining this methodology as it is still limited in its use in Scandinavian countries, e.g. in Sweden. This is partly due to historical-practical reasons (regular ultrasound training is not included in the neurology specialization), partly due to insufficient study data from other parts of the world and partly due to lack of data from a Scandinavian cohort. Until now, there are no published data on the diagnostic value of SN+ as biomarker for PD versus other Parkinsonian syndromes neither in a Swedish nor Scandinavian population.

After establishing cut off values in a healthy cohort, the results of the present study support the use of TCS for diagnosing PD. We examined the correlation between TCS findings and the finally established clinical diagnosis after a reasonably long FU period. After almost 8 years, we found a rather limited sensitivity (75 %) compared to some previous studies, where sensitivity exceeded 80 % [23,24], but found good specificity (80 %) and an even higher PPV (90 %) with similar findings in specified subgroups including revised PD-patients, patients who have been examined by TCS early and those who went through PET/SPECT scans. Our findings correspond to two comprehensive *meta*-analyses: Pooled data from 15 original studies found 75 % sensitivity and 69 % specificity when comparing PD vs. APS [25], while a more recent *meta*-analysis of 18 studies with quite some overlap between the studies (almost the same original trials included, n = 14) demonstrated 77 % sensitivity, 81 % specificity and 90 % PPV [24]. The most recent *meta*-analysis of the 9 most recent trials (with some overlap, n = 2) found a 85 % sensitivity and 71 % specificity, PPV was not reported [23].

The patient cohorts studied by TCS so far were predominantly from Central/Southern and Eastern Europe, South America, and East Asia. There is evidence that lighter skin pigmentation is associated with elevated SN echogenicity in a geographically defined population [26,27]. However, in a direct comparison using the same ultrasound device, the echogenicity of SN and the frequency of SN+ were similar in Central European and East Asian populations [28]. There are differences mainly in the frequency of a sufficient transcranial bone window between Caucasian and Asian subjects, with the latter having a lower percentage of adequate transcranial bone windows, e.g., 25 % dropouts in a larger Chinese cohort, but SN positivity in TCS could still effectively differentiate PD from ET, PSP, and MSA in this Chinese population [29]. In patients with MSA, PSP and ET, SN is mostly normal that means that SN+ is found in only 10 % of cases or less [4,13,17,18,25,30]. On the other hand, there is some evidence that the SN+ feature cannot distinguish between PD and CBD patients due to almost the same amount of positive findings in both groups [18,24,31]. Three recent *meta*-analyses concluded that SN+ as sonological biomarker can differentiate between PD and APS with a sensitivity of 75–85 % and a specificity of 70–81 % [[23], [24], [25]].

To improve the differential diagnosis of PD vs. APS using TCS, the sonographic assessment also of lenticular nucleus has been recommended [4,18]. By including other findings on TCS, e.g. the hyperechogenicity of basal ganglia and an increased third-ventricle width, characteristic patterns of TCS correspond to different Parkinsonian syndromes (MSA, PSP, CBD) facilitating differential diagnostics [4,16,18]. Lenticular nucleus hyperechogenicity is not only indicative of APS, but also a typical finding in Wilson‘s disease or in distinct iron storage diseases [7,[32], [33], [34]]. TCS of lenticular nucleus, however, requires special training of the examiner [18] and was not part of the protocol of the present study. It should be noted that SN+ occurs more frequently in copper and iron storage diseases, which supports the recommendation of a combined assessment of SN, basal ganglia, and ventricles for the purpose of differential diagnosis [7,34,35]. Nowadays, in our labs the combined assessment of SN, lenticular nucleus and 3rd ventricle on TCS is performed for the discrimination of parkinsonian disorders. Still, for distinct diagnostic questions (e.g. the differentiation between essential tremor and PD), the assessment of SN is sufficient [18].

The strength of the present study includes the long FU-time of 7.9 years in a Swedish population that to our knowledge has not yet has been investigated. Although definite diagnosis in neurodegenerative disorders only can be obtained by neuropathological assessment on autopsy, a long FU period increases the likelihood for correct clinico-pathological correlation. In addition, the clinical diagnoses were independently validated by an experienced movement disorder specialist unaware of the SN+ biomarker status.

However, there are limitations to the present study design: small sample size, retrospective design and possible selection bias with not all parkinsonism patients included from initial diagnostic evaluation are issues to be considered. There was no formal blinding regarding the initially suspected diagnoses at the time when TCS investigations were performed. However, the considerable number of patients with change of the diagnosis during follow-up should have reduced the potential bias caused by initial unblinding of the sonographer.

The rather high overall rate of diagnostic revisions (52 %) in our study can be explained by the fact that patients with nonspecific early parkinsonism who did not meet the clinical MDS society criteria for PD were included initially. The previously reported error rates of approximately 10–20 % for diagnoses in movement disorder centres usually refer to patients who were originally diagnosed with PD. Looking only at the patients in our cohort who were originally diagnosed with PD (n = 36), the diagnostic error rate is within the reported range (n = 7; 19 %). The higher number of overall diagnostic revision (52 %) itself could partially be explained by an initial use of the category *unspecified MDS* (ICD-10-codes: G25.9 or G23.9) in early parkinsonism patients not yet fulfilling the clinical criteria for PD. If one only counts the final PD-patients who got initial preliminary diagnosis ET or “unclear syndrome/no MDS” and view 8 patients with unspecified parkinsonism suggestive for PD as initially suspected PD, the diagnostic revision rate after FU drops to 27 % of all true PD-patients.

The time gap between 1st visit and TCS examination (7.5 months in mean) was most likely not of importance due to the previously demonstrated stable SN status over time [8]. The SN echogenic sizes found here in PD patients are within the same range as reported recently by Chau et al., who applied the same ultrasound system [21]. The absolute differences between PD and non-PD patients regarding bilateral SN size were small, probably because the original validated cut-off value (0.23 cm^2^) was meant to differentiate PD from healthy controls and not from other basal ganglia disorder. These study findings may be due to a heterogeneous control group (non-PD incl. 8 APS, 7 secondary/VP, 3 undefined and 2 ET) with mixed diagnoses which were harder to differentiate from PD by SN+ only. It has been hypothesized from one earlier study by Behnke et al. that a mild to moderate SN hyperechogenicity is frequent in patients with unspecified APS [36].

We did not reach the high levels of sensitivity and specificity reported in some previous studies [9], which is probably due to the assessment of SN only (instead of assessing also the lenticular nucleus), the rather small sample size, and a different patient selection. One might argue that a second TCS examination of our patients could have increased the diagnostic accuracy. Until now, digitized measurements of SN echo-intensity by computerized algorithm were not clearly superior to manual planimetric measurement according to internationally agreed recommendations by experienced ultrasonic experts [10,37], even if some previous trials have postulated increased sensitivity for PD compared to healthy and individuals with Wilson‘s disease by using the recent TCS-MRI-fusion imaging technique [38]. The recently developed real-time TCS-MRI fusion imaging technique could potentially increase the detection rate of SN+ and thereby improve the differentiation between PD and APS [38]. Moreover, planimetric measures and digitized echo-intensity measures have been proposed to capture different pathologies causing SN+, which could explain why they may differ to some degree [39]. Therefore, an adequate reliability of the planimetric standard measures has been proposed being achievable by using strict protocols, standardized documentation and well-trained neurosonologists [40] which we strictly followed.

The TCS technique needs a sufficient degree of training before one is able using it on a regular basis [4]. Therefore, in the recent update of the German guideline on PD, the education requirements to be regarded as qualified TCS investigator in PD have been outlined [18], which could be worthwhile to adapt to the national guidelines in other European countries. Another issue is the dependence of TCS on sufficient temporal bone windows for insonation in patients or probands. Adequate bone window could be seen in studies at ≥90 % of patients/healthy probands, in the present study we assessed even 96 % of participants with satisfying bone window.

Considering the available neuroimaging techniques, TCS offers the advantages of non-invasiveness, easy repeatability, and less interference from patient movement. Even though conventional MRI can detect structural differences between PD and APS – such as the putaminal rim or hot cross bun sign in MSA or the hummingbird sign in PSP – these are generally only signs of a later stage of the disease and do not help to distinguish between patients with early-stage or mild PD. [41]. Presynaptic molecular imaging using SPECT does not allow differentiation between PD and APS at any stage of the disease [41,42]. Similar to SPECT, presynaptic ^18^F-Dopa PET shows reduced tracer uptake in both PD and APS, without it being able to differentiate between the two [42]. Hellwig et al. conducted a comparison between TCS and diagnostic molecular imaging (FDG-PET) in 36 patients, which showed a significant correlation between the FDG-PET and TCS findings in distinguishing between different types of Parkinsonian disorders [13]. The sensitivity/specificity for the diagnosis of APS using FDG-PET was 82 %/100 % and using TCS 82 %/85 %. The diagnostic accuracy did not differ significantly between the modalities in this study (p = 0.63) [13].

In conclusion, our findings in a Swedish cohort support the use of TCS as a valuable add-on tool for the diagnosis of PD in a group of patients with parkinsonism at a tertiary referral center, although the overall need of achieving an reliable individual prediction may remain unmet by using TCS of SN as stand-alone test. However, TCS can be generally regarded as a non-invasive, cost-effective and complementary diagnostic method, provided that it is performed by a qualified investigator. The finding of SN+ in a patient with suspected PD can mostly avoid the use of expensive nuclear medicine examinations, whereas a negative result does not rule out PD, requiring TCS of other brain structures, or additional diagnostic workup to discriminate PD from other parkinsonian disorders. TCS of basal ganglia and especially prospective head-to-head studies comparing TCS with other (nuclear medicine) diagnostic methods in a Swedish cohort would be of great interest. To implement TCS in clinical practice, it is crucial to keep a good sonographer’s competence, a structured examination protocol including documentation and to limit the use of TCS to the clinical condition of parkinsonism.

## CRediT authorship contribution statement

**M. Stiehm:** Writing – review & editing, Writing – original draft, Visualization, Validation, Project administration, Methodology, Investigation, Funding acquisition, Formal analysis, Data curation, Conceptualization. **C. Nilsson:** Writing – review & editing, Visualization, Supervision, Resources, Methodology, Conceptualization. **Ö. Skogar:** Validation, Investigation. **U. Walter:** Writing – review & editing, Supervision, Methodology, Formal analysis, Conceptualization.

## Funding

This work was supported by The Husbands Stoltz‘ Foundation, Malmö and The Elsa Schmitz‘ Foundation, Lund.

## Declaration of competing interest

The authors declare that they have no known competing financial interests or personal relationships that could have appeared to influence the work reported in this paper.
