# Supplementary material for: The diagnostic value of transcranial sonography in Swedish parkinsonism patients: A retrospective cohort study with long-term follow-up
Source: Clin Park Relat Disord. 2025 Dec 6;13:100411. doi: 10.1016/j.prdoa.2025.100411 (PMC12754217; doi:10.1016/j.prdoa.2025.100411)
Supplement: Supplementary Data 1 [file mmc1.docx]

**TRANSPARK_LONG**

**Supplementary Material**

**Table 3**

Values of the Substantia Nigra Hyperechogenicity (SN+) finding by Transcranial Sonography (TCS) in predicting the diagnosis of Parkinson`s Disease (PD) in our cohort (n=71)

|  | Value | 95% CI |
| --- | --- | --- |
| Sensitivity | 74.51% | 60.37% to 85.67% |
| Specificity | 80.00% | 56.34% to 94.27% |
| Positive Predictive Value (*) | 90.48% | 79.58% to 95.86% |
| Negative Predictive Value (*) | 55.17% | 42.31% to 67.38% |
| Accuracy (*) | 76.06% | 64.46% to 85.39% |

**Table 4**

Preliminary diagnosis at 1^st^ visit vs. most certain (final) diagnosis after follow-up (FU)

| Pre-liminary diagnosis at 1^st^ visit |  | Final diagnosis after FU | | | | | |  |
| --- | --- | --- | --- | --- | --- | --- | --- | --- |
|  |  |  | PD | APS | ET | Sec. incl. VP/unspec | No MDS | **Total** |
|  | PD |  | 29 | 5 | 0 | 2 | 0 | **36** |
|  | APS |  | 0 | 0 | 0 | 0 | 0 | **0** |
|  | ET |  | 7 | 0 | 2 | 1 | 0 | **10** |
|  | Sec./unspec. |  | 8 | 2 | 0 | 6 (3+3) | 0 | **16** |
|  | No MDS |  | 7 | 1 | 0 | 1 | 0 | **9** |
|  | **Total** |  | **51** | **8** | **2** | **10** | **0** | **71** |

*Abbreviations: Follow-Up (FU); Parkinson`s disease (PD); Atypical Parkinsonism (APS); Essential Tremor (ET); vascular parkinsonism (VP); movement disorder (MDS).*

**BONUS Figures A+ B**

**[Only captions, full size images are uploaded separately]**

Figure A: The butterfly-shaped midbrain transection is highlighted by a yellow line surrounding the midbrain. The red line indicates the ipsilateral hyperechogenic Substantia nigra (SN) and is enlarged in this case (PD patient).

**
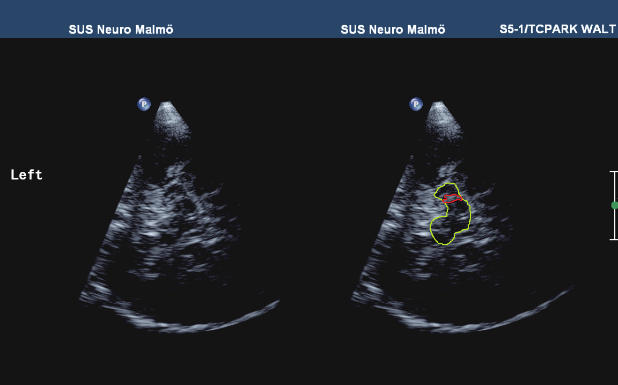
**

Figure B: The butterfly-shaped midbrain transection is highlighted by a yellow line surrounding the midbrain. The red line indicates a normal-sized ipsilateral hyperechogenic Substantia nigra (SN) from a healthy individual.

**
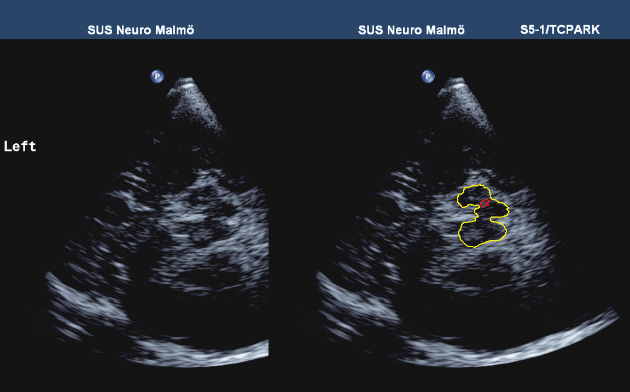
**
