# Supplementary figures and images for: The diagnostic value of transcranial sonography in Swedish parkinsonism patients: A retrospective cohort study with long-term follow-up
Source: Clin Park Relat Disord. 2025 Dec 6;13:100411. doi: 10.1016/j.prdoa.2025.100411 (PMC12754217; doi:10.1016/j.prdoa.2025.100411)

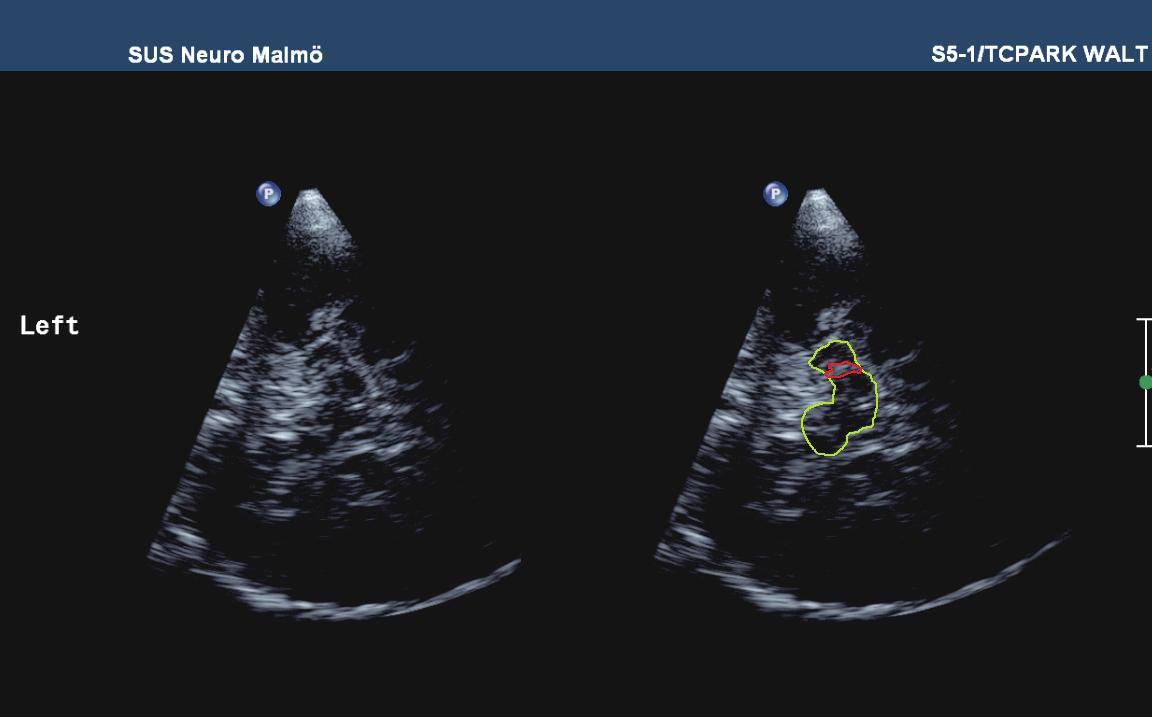

Supplement: Supplementary Figure A [file mmc2.jpg]

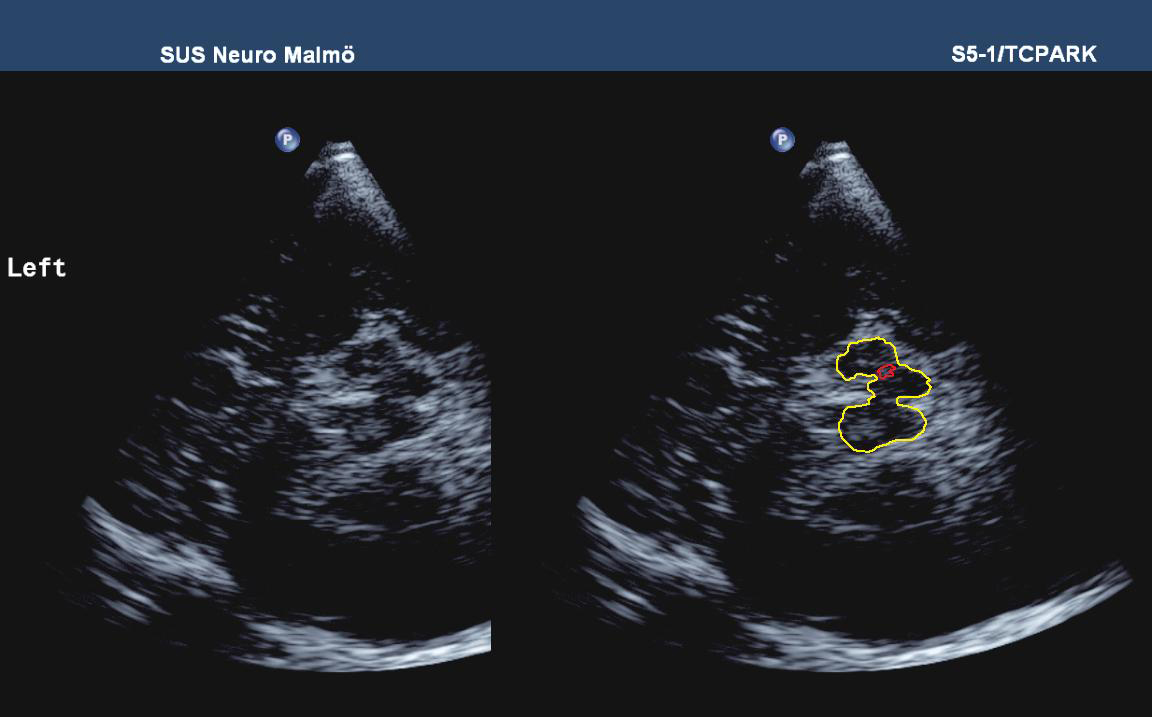

Supplement: Supplementary Figure B [file mmc3.jpg]
